# Application of *Bother* in patient reported outcomes instruments across cultures

**DOI:** 10.1186/1477-7525-12-18

**Published:** 2014-02-13

**Authors:** Mary C Gawlicki, Shawn M McKown, Matthew J Talbert, Barbara A Brandt

**Affiliations:** 1 Corporate Translations Inc, 77 Hartland Street, East Hartford, CT 06108, USA; 2 Linguistic Validation, Corporate Translations Inc, 77 Hartland Street, East Hartford, CT 06108, USA; 3 Linguistic Validation, Corporate Translations Inc, 2039 W. Wabansia Ave., Chicago, IL 60647, USA

## Abstract

**Background:**

The objective of this study was to determine the applicability of the term *bother,* as used in Patient Reported Outcomes (PRO) instruments that will be translated into foreign languages from English for the United States. *Bother* is versatile in English for the U.S., in that it can describe negative mental states and physical sensations, as well as social disturbances. *Bother* has many different meanings across cultures, due to this versatility. Alternatives for *bother* were explored for future PRO instrument development.

**Methods:**

A PRO instrument used to evaluate the degree of *bother* resulting from psoriasis was analyzed. This disease can negatively impact patients physically, emotionally and socially. Translations of *bother* were analyzed to determine its meaning when translated into other languages. Cognitive debriefing was conducted on psoriasis patients with the instrument containing *bother.* Following cognitive debriefing, a questionnaire was distributed to linguists and cognitive debriefing subjects to collect definitions of *bother* in each target language, and detail any difficulty with translation.

To establish alternatives to *bother* and demonstrate the breakdown of concepts within *bother*, translations of the Dermatology Quality of Life Index (DLQI) were analyzed. This instrument was selected for its focus on psoriasis and use of terminology that lacks the ambiguity of *bother*.

**Results:**

An analysis of back-translations revealed that *bother* yielded a back-translation that was conceptually different from the source 20% of the time (5/26). Analysis of alternative terminology found in the DLQI revealed much greater conceptual equivalence when translated into other languages.

**Conclusion:**

When developing the wording of PRO instruments, the terminology chosen should be applicable across languages to allow for international pooling and comparison of data. While all linguists and subjects of cognitive debriefing understood *bother* to have a negative connotation, a large variety of definitions and synonyms provided across languages showed a lack of conceptual equivalence. Ambiguity of the term across cultures may result in variation in translation, impacting subsequent international data pooling. Analysis of alternatives revealed that measurement of unambiguous terminology produces the greatest conceptual equivalency across languages and cultures.

## Background

Any report of the status of a patient's health condition that comes directly from the patient, without interpretation of the patient's response by a clinician or anyone else, has been termed a patient-reported outcome (PRO) [1]. Dependent upon the disease or condition of interest, a PRO may be the sole source of data from which drug efficacy can be measured, while in others it may provide supplementary information on how the disease and its treatment impact patients’ functioning and feeling. PROs are collected in clinical trials via standardized questionnaires designed to measure an explicit construct such as symptoms, activity limitations, health status, health-related quality of life (HRQOL). The use of PROs as clinical trial endpoints continues to be widespread, with over 45% of all New Molecular Entity (NME) or Biologics License Applications (BLA) submissions between 2006 and 2010 utilizing these instruments in some capacity [2,3].

There are many challenges when PROs are included in multinational clinical trials [4]. One challenge is to ensure that terminology is translated and understood in the manner intended. In order to achieve cultural equivalence, PRO instruments must be translated using the appropriate methodology. When PRO instruments are utilized in a multinational clinical trial, they must undergo translation and linguistic validation to ensure conceptual equivalence and proper adaptation for the target language and country. This process requires translation by two independent translators and subsequent reconciliation to reach consensus, creating a “harmonized” translation. A third translator then “back-translates” the text back into English [5]. The back-translation is then reviewed to ensure conceptual equivalence between the translation and source text. Upon completion of this review, in-country cognitive debriefing interviews are carried out with local subjects located by a recruiter living in the target country. The purpose of these interviews is to test subject comprehension and readability of the translated text, and to make necessary revisions to ensure and maximize conceptual equivalence and cultural appropriateness. Failure to maintain conceptual equivalence calls the validity of collected data into question and impedes international data pooling [6].

*Bother* is commonly used in PRO instruments to measure a combination of patient satisfaction and discomfort with the disease and/or treatment. Although generally understood to have a negative connotation, *bother* can be used to describe physical, emotional and social states in U.S. English. This ambiguity results in a wide range of definitions and interpretations across many languages and cultures. Achievement of conceptual equivalence, therefore, becomes much more difficult, possibly threatening the data validity and pooling in multinational clinical trials [7]. The purpose of this research is to explore the meaning of *bother* when translated into different languages, and to determine the degree of difficulty in achieving conceptual equivalence. Following the analysis of *bother* when translated, acceptable alternatives were explored for use in PRO instruments.

## Review of current literature

The findings of a previous observational study of *bother* were analyzed. Its purpose was to find contexts in which *bother* is used in US English and to form a foundational definition. 2000 statements that included the word *bother* were randomly selected from a website called LiveJournal.com. This site contains many users’ public diaries in the form of an online blog entry on a topic of the writer’s choice. The results of this observational study revealed that *bother* was used to refer to negative emotional feelings, pain in a physical sense, and preoccupation in one’s own thoughts [8].

In a study to measure erectile dysfunction due to benign prostatic hyperplasia, subjects were asked to assess their ability to obtain an erection and their satisfaction with their ejaculation [9]. Additionally, subjects were asked to assess the level of *bother* associated with their erectile ability and ejaculatory dysfunction. The *bother* assessment for both erectile and ejaculatory dysfunction did not seem to yield additional useful data for assessment of erectile dysfunction. It was also found that throughout the 24-week study, the *bother* score remained stagnant, while the erection and ejaculatory assessment changed throughout the course of the study.

In a study of lower urinary tract symptoms, a PRO instrument was developed which utilizes *bother* to assess severity of symptoms [10]. During the development of the PRO instrument, *bother* proved to be difficult to measure, due to its many “widespread interpretations.” In order to create clarity for the respondent and to improve data validity and pooling, the PRO instrument was developed with *bother* appearing in association with specific lower urinary tract symptoms, such as patient’s work productivity impact, mental health and sexual health. Although it was not clear whether data validity improved as a result, this alleviated the issue of conceptual ambiguity.

Solutions to ambiguous terminology, such as *bother,* have been observed in previous studies*.* A translatability assessment [11] was applied to *distress*, which was hypothesized to have different meanings in other languages and cultures [12]. *Distress* was used in the Hospital Anxiety and Depression Scale (HADS), a questionnaire used to evaluate a patient’s views of cancer treatment taking place in Israel, therefore requiring that the instrument be translated into Hebrew and Arabic. Because *distress* is perceived differently between the two languages, the developers surveyed psychologists, social workers and stress researchers who were fluent in Hebrew and Arabic in an effort to create a definition of *distress* compatible with both languages. Respondents were asked to define *distress* in their own language. There was no consensus on a definition for *distress* among those surveyed. However, the solution was to create two distinct definitions of *distress*: *stress from anxiety*, and *stress from depression*. This resulted in two questions broken down into more specific measures: one concerning a patient’s anxiety with regard to their cancer and treatment, and another concerning a patient’s depression resulting from their cancer and treatment.

## Methods

In the psoriasis PRO instrument analyzed as part of this study, patients were asked to what degree their symptoms *bothered* them. The name of the PRO questionnaire, its developer and owner must remain strictly confidential, per contractual agreement. Questionnaire data and key terms have been presented and analyzed as appropriate to honor this agreement. It should be noted that psoriasis was the focus for this study, as the disease may cause physical, emotional and social adverse affects. The PRO instrument went through linguistic validation – the process of forward and back-translation, followed by in-country cognitive debriefing. After completing the linguistic validation, cognitive debriefing data concerning the translation of *bother* were analyzed.

Translation and linguistic validation [13] of the psoriasis PRO instrument using *bother* within a Numeric Rating Scale (NRS) was carried out in 26 languages: Arabic (Israel), Bulgarian (Bulgaria), Czech (Czech Republic), Danish (Denmark), Dutch (Belgium), Dutch (Netherlands), English (Canada), English (Israel), English (UK), French (Belgium), French (Canada), German (Austria), German (Germany), Hebrew (Israel), Hungarian (Hungary), Italian (Italy), Japanese (Japan), Norwegian (Norway), Polish (Poland), Romanian (Romania), Russian (Israel), Russian (Russia), Spanish (Colombia), Spanish (Mexico), Spanish (Spain) and Spanish (US). Translatability and interpretation of *bother* were explored through analysis of linguistic validation data. Additionally, linguists and cognitive debriefing subjects were surveyed to determine difficulties in translating the instrument and in defining *bother*.

Although *bother* can be utilized across multiple parts of speech in English, as a noun, verb, or adjective, only the adjectival form is utilized in the observed PRO instrument (“How *bothered* are you….”). As a result, all observed translations, back-translations, and cognitive debriefing results refer exclusively to one consistent part of speech. In order to gather feedback on foreign-language use of *bother* across multiple parts of speech, the follow-up survey provided to linguists and cognitive debriefing subjects asked respondents to define and provide comment on the adjectival form found in the instrument and the term *bother* itself, which may function as either a noun or a verb. Upon review and comparison of the provided definitions and responses, it was determined that the reported meanings of *bother* showed no meaningful difference across observed parts of speech in non-English languages.

Following linguistic validation, all back-translations and cognitive debriefing data were analyzed, with a focus on the translation of *bother*. The dictionary definition of *bother* was compared to the dictionary definition of the English back-translation, and discrepancies were recorded. *Skin* was analyzed as a control term for comparison. After linguistic validation was completed, a voluntary questionnaire was sent out to all linguists and cognitive debriefing subjects. Linguists were asked to provide the term they selected for *bother* in their language, how they defined *bother* in their translation, and to elaborate on any translation difficulties. Subjects were asked how they defined *bother* and in what contexts it may be used in their language.

It is theorized that including more specific terminology which identifies the adverse effects of a disease individually will yield greater conceptual equivalency when translated. Examples pertaining to psoriasis were found within the Dermatology Quality of Life Index (DLQI) [14]. The follow list shows items found in the DLQI that avoid the use of *bother*.

• Over the last week, how *itchy, sore, painful or stinging* has your skin been?

• Over the last week, how *embarrassed or self conscious* have you been because of your skin?

• Over the last week, how much has your skin *interfered* with you going shopping or looking after your home or yard**?**

• Over the last week, how much has your skin *influenced* the clothes you wear?

• Over the last week, how much has your skin *affected* any social or leisure activities?

• Over the last week, has your skin *prevented* you from working or studying?

• Over the last week, how much has your skin *created problems* with your partner or any of your close friends or relatives?

• Over the last week, how much has your skin caused *any sexual difficulties*?

© Dermatology Life Quality Index. A Y Finlay, G K Khan, April 1992.

To test the hypothesis that more specific terminology will yield greater conceptual equivalency in translation, the linguistic validation results of the selected terminology were compared to those of *bother.* The example used to test this hypothesis will be the DLQI.

The subjects of cognitive debriefing were volunteers who self-reported their psoriasis and gave consent to participate in the interview. Confidentiality was protected as subject names were not collected nor linked to any of the data they provided. No medical data was collected. Subject contributions were solely for linguistic research and to ensure comprehension of the translated text. Additionally, the manner in which participant data was collected was determined to be ethically acceptable by the Institutional Review Board Services of Aurora, Ontario in Canada.

## Results

### Back-Translation Analysis

An analysis of all harmonized translations of the psoriasis instrument containing *bother* showed that the term was back-translated as something conceptually different from the source 19% (5/26) of the time. Examples of non-equivalent back-translations were *discomfort, embarrassment* and *anxiety*. Words that appeared most frequently as back-translations of *bother* and their respective definitions are as follows:

• *Trouble*: To disturb mental calm and contentment

• *Annoy*: To disturb another in a way that displeases, troubles or slightly irritates

• *Disturb*: To interrupt quiet, rest, peace or order

• *Discomfort*: Absence of comfort or ease, uneasiness, hardship or mild pain [15]

With the exception of *discomfort*, all of the most frequent back-translations were conceptually similar to *bother* and therefore contain the same level of ambiguity. The most significant back-translation outlier for *bother* was *anxiety. Anxiety* was observed to be the least conceptually similar to *bother*, as it refers to *nervousness* or *agitation. Anxiety* was the selected Russian back-translated term for *bother* in both Russia and Israel.

### Cognitive Debriefing Analysis

Following forward and back translation, the psoriasis instrument underwent cognitive debriefing interviews with five subjects per country. The exception was Japanese for Japan, where only four subjects were debriefed, as recruiters were unable to find a fifth volunteer with psoriasis. Subjects had been diagnosed with psoriasis and were diverse as to age, gender and level of education. The Table 1 displays a breakdown of the cognitive debriefing sample. Suggested replacements or deletions of the translated word for *bother* by the subjects are summarized in Table 2.

**Table 1:** Summary of characteristics of those who took part in cognitive debriefing

**n = 129**		
Gender		
Males: n = 64		
Females: n = 65		
	Age (years)	Education (years)
Average	47.2	13.2
Median	47	13
Standard deviation	14.9	3.2
Minimum – Maximum	18 – 84	6 – 24

**Table 2:** Notable issues with bother found during debriefing

**Arabic (Israel)**	**Two subjects suggested deleting the term “مضايقتك” ( *bothered *), since it is very similar in meaning to **انزعاجك” **( *troubled *). One suggested replacing it with منزعج (upset) throughout the questionnaire.**
English (UK)	One subject was confused as to whether *bother* referred to physical or mental *bother*.
French (Canada)	One subject did not understand the word “l’importunité” (*bothersomeness*) and suggested using “le dérangement” (*bothersomeness*) instead.
German (Austria)	One subject noted that he makes no distinction in meaning between the words “störend” (*bothered*) and “lästig” (*troubled*).
German (Germany)	One subject suggested deleting “störend” (*bothered*) from one question.Two subjects would replace “störend” (*bothered*) with “belastend” (*troubled/stressed*).
Italian (Italy)	One subject suggested replacing “misura del fastidio causato” (*bothersomeness measure*) with “valore del malessere dovuto” (*discomfort value*) while another suggested replacing “fastidio” (*bothersomeness*) with “turbamento” (*bothersomeness*). One subject suggested replacing “infastidiscono” (*bothered*) with “infastidisce” (*bothered*); another one suggested replacing “infastidiscono” (*bothered*) with “disturbano” (*disturbed*).
Spanish (Spain)	Two subjects suggested using only “molestias” (*bothered*) and not “preocupación” (*troubled*).

Several anomalous findings resulted from cognitive debriefing analysis. During cognitive debriefing, one German subject suggested removing *bother* without replacement, and noted the German word for *bother* (“stören”) to be a “weak term”. Similarly, a subject in the United Kingdom was uncertain as to whether *bother* was to be used in a mental or physical context. This is significant in a psoriasis PRO, as the disease may cause physical pain resulting from scaling or abrasions on the skin, or mental anguish due to the skin’s unsightly appearance. In this case, due to the ambiguity of *bother*, it is not clear whether the severities of mental or physical symptoms of psoriasis are being measured.

Although *bother* was understood and paraphrased correctly by all subjects, the dominant issues raised were widespread discrepancies of interpretation between subjects. This variation in meaning is explored below.

### Analysis of linguist and subject questionnaire responses

All linguists were asked to provide the term they selected for *bother* in their language, and explain how the term is interpreted in their language and country. Additionally, linguists were asked to provide synonyms in English for their selected translation of *bother* and note whether they had difficulty achieving conceptual equivalence in their language. Not all linguists involved in the linguistic validation project are represented in the questionnaire results, as participation was voluntary. Table 3 presents a summary of the responses of those who participated.

**Table 3:** Summary of linguist responses to the questionnaire

**Language (Country)**	**Term selected in target language**	**Definition of *bother *** **in target language**	**Difficulty with translation**
Bulgarian (Bulgaria)	безпокоя се	Troubled, annoyed, worried, inconvenienced.	There are too many definitions of this concept in Bulgarian.
Czech (Czech Republic)	obtěžovat	Something that makes one feel unpleasant or uncomfortable.	None noted.
Danish (Denmark)	Gene	Simply means something that *bothers* you.	None noted.
Dutch (Belgium)	Last	Annoyance or something that makes one feel uncomfortable, or even causes pain. The term can also describe something that is chronic.	None noted.
French (Belgium)	Gene	Being troubled, annoyed (could be physical), embarrassed.	*Bother* could mean “annoyance” or “worry” in English and no French word.
French (Canada)	Déranger	Disrupt, introduce a change, or make something different in way that is not pleasant.	Translation was found to be very difficult because the term is general in meaning.
German (Austria)	Stören	Irritating or disturbing.	Difficult to find a German equivalent.
German (Germany)	Störend	To be a nuisance.	There are a wide variety of meanings and associations for *bother*.
Hebrew (Israel)	מוטרד	To worry, be anxious or uneasy.	No difficulty translating.
Hungarian (Hungary)	zavar	To disturb, distract or interfere with normal life.	Unable to determine an exact equivalent.
Italian (Italy)	Fastidio	The degree to which something may trouble or the way one might dislike something. It may also mean annoyed, irritated or aggravated.	Was slightly difficult, but the selected term best conveys the meaning.
Japanese (Japan)	悩む	Physical or psychological suffering.	No, as Japanese is a vague language, so it was not difficult to find another vague term.
Norwegian (Norway)	Plage	Someone or something that temporarily is disturbing or troubling someone.	No issues translating.
Polish (Poland)	Dokuczać	To be troublesome or a nuisance.	No equivalent term in Polish to encompass all that *bother* means in the context of mental and physical issues.
Romanian (Romania)	a deranja	To cause discomfort, inconvenience, embarrass, disrupt or trouble.	No issues translating. Found the only acceptable term.
Russian (Israel)	беспокоить	To disturb, worry or trouble.	No difficulties translating. Found the best term to convey the intended meaning of *bother*.
Russian (Russia)	Беспокоить	To cause anxiety, inconvenience, or trouble.	Yes, because English has more synonyms than Russian.
Spanish (Mexico)	molestar	It is a colloquial term used by doctors to ask patients what *bothers* them.	No. Selected term correlates with the meaning in English.
Spanish (Spain)	molestia	Sensation of discomfort or *bother*.	Yes, as it was difficult to pick one of many possible terms to convey the entire meaning of *bother*.

It was observed that the definition of *bother*, when translated, varies among different languages and cultures, accounting for the variance in understanding among subjects. For example, the Dutch term for *bother* describes something that is *constant* or *chronic*, while the Norwegian term refers to something that is *temporary*. In another example, the terms selected in French for Canada and French for Belgium differed conceptually. The definitions provided by linguists were “to disrupt or make a change that is not pleasant” in French for Canada, and “to be troubled, annoyed or embarrassed” in French for Belgium. Overall, linguists reported issues with translating *bother.* Four out of a sample of 19 linguists attributed translation difficulties to the ambiguity of the term. Three out of this same sample of 19 linguists attributed translation difficulties to the fact that the target language has no direct translation for *bother*.

Subjects’ questionnaire responses revealed that the majority of subjects within a single language-country pair had common interpretations of *bother*. For example, all Bulgarian for Bulgaria subjects defined *bother* as *troubled and uneasy*, while all French for Belgium subjects defined it as *embarrassment*. In some cases, the same language validated in multiple countries yielded similar interpretations. For example, all Dutch-speaking subjects in both the Netherlands and Belgium interpreted *bother* as a chronic hindrance and *something that is always there*. Additionally, a majority of German-speaking subjects from Germany and Austria interpreted *bother* as *discomfort*. On the contrary, there were several instances in which a common language yielded different results for each country. While all Spanish for Mexico subjects interpreted *bother* as *a provocation of anger*, a majority of Spanish for Colombia subjects interpreted *bother* as *discomfort*. Furthermore, all English for Canada subjects defined *bother* as *difficulty* or *trouble*, while all English for the United Kingdom subjects defined it as *to irritate* or *to interrupt*.

## Discussion

A review of the four languages in this study for Israel (Hebrew, English, Arabic and Russian) revealed interesting results. As was the case with all subjects in the study, all Israeli subjects were asked to define *bother* as used in the target language for cognitive debriefing, as several of the subjects spoke a different native language, or were bilingual. Table 4 shows a breakdown of the native languages of each subject, in addition to other languages they speak fluently. English-speaking Israeli subjects did not conform to a common definition of *bother*. This could be attributed to the fact that English was not their native language, and regional differences in terminology. Definitions provided by a sample of four English-speaking Israeli subjects are as follows:

• To make anxious

• To cause inconvenience and disturbance

• To interfere and annoy

• To make problems

**Table 4:** Languages of the Israeli subjects

**Language**	**Native language**	**Other languages known by the subject**
Arabic (1)	Arabic	none
Arabic (2)	Arabic	none
Arabic (3)	Arabic	none
Arabic (4)	Arabic	none
Arabic (5)	Arabic	none
English (1)	Russian	Hebrew and English
English (2)	Russian	English, Hebrew and Ukrainian
English (3)	Russian	Hebrew, English, Arabic
English (4)	English	Hebrew
Hebrew (1)	Hebrew	None
Hebrew (2)	Serbian	Hebrew
Hebrew (3)	Hebrew	none
Hebrew (4)	Romanian	Hebrew
Hebrew (5)	Russian	Hebrew
Russian (1)	Russian	Hebrew
Russian (2)	Russian	Hebrew
Russian (3)	Russian	Hebrew
Russian (4)	Russian	Hebrew

During cognitive debriefing interviews, subjects for Hebrew, Russian and Arabic for Israel reported a common interpretation of *bother*. On the contrary, linguists for these languages did not share a common interpretation of *bother*, as demonstrated below:

• Hebrew – Negative connotation (that can be used in a number of contexts)

• Russian – Disturbance

• Arabic – Discomfort

Despite having a native tongue other than the questionnaire’s target language, or bilingualism, a discrepancy in the interpretation of *bother* is still apparent in the results, indicating that a level of *bother* is a problematic measure when attempting to pool data across residents of Israel.

Terminology found in the DLQI analyzed as an alternative to *bother* revealed that more specific terminology will yield greater conceptual equivalency. The back-translated English words of the specific terms were compared to the source English words of the DLQI, mirroring the analysis used for *bother*. It was often observed that the specific terminology found in the DLQI back-translation was identical to the source. There were a few instances where the back-translated word was different from the English source, but still conceptually equivalent. Examples of such are *interfered* back-translated as *prevented* or *disrupted*, and *self-conscious* back-translated as *ashamed.* Table 5 compares the achievement of conceptual equivalence of specific alternative terminology as compared to *bother*. There were 22 available DLQI translations for analysis and 26 translations of the psoriasis instrument containing *bother* were available for analysis.

**Table 5:** **Achievement of conceptual equivalence of DLQI concepts vs. *bother***

**DLQI question concept**	**Percentage of back-translations conceptually equivalent to source n = 22**
*Itchy, sore, painful or stinging*	100%
*Embarrassed or self conscious*	95.5%
*Interfered*	100%
*Influenced*	100%
*Affected*	100%
*Prevented*	95.5%
*Creation of problems*	100%
*Causing difficulty*	100%
***Bother *** **question concept**	**n = 26**
*Bothered, bothersomeness* (as related to scaling and flakiness, skin thickening, skin discoloration or redness)	80%*

*Different language/country pairs from DLQI.

As shown in Table 5, the occurrence of achieving conceptual equivalence with translation of the more specific terminology was at least 95.5% (21/22) in all cases. Examples of back-translations that were not equivalent were *self-conscious* back-translated as *shy* and *prevented* back-translated as *caused a problem.* Unlike *bother,* however, those issues were corrected easily after consultation with the linguists.

## Conclusion

In development of PRO instruments, the terminology chosen should be applicable across languages to allow for multinational data pooling and comparison. The varied definitions of *bother*, as provided by linguists and subjects with psoriasis in response to the questionnaires, demonstrate that the term does not meet these criteria, and does not maintain conceptual equivalence across languages and countries when translated. Furthermore, as demonstrated by the findings within the four languages of Israel, *bother* may also present a lack of conceptual equivalence between languages within a single country. As further demonstrated by this research, the use of more specific concepts as an alternative of *bother* will yield greater conceptual equivalence across languages. To conclude, the use of *bother* in PRO instruments will threaten the validity of data and hinder data pooling from multinational studies. It is recommended that *bother* be avoided when developing PRO instruments intended for multinational clinical trials.

## Competing interests

The authors declare that they have no competing interests.

## Authors’ contributions

MT conducted analyses of the linguistic validation results, conducted analyses of the questionnaire results, and drafted the manuscript. BB designed the study, created the questionnaires and made significant revisions to the first draft of the manuscript. All authors read, made revisions to and approved the final manuscript.
